# Patterns of Loss and Regeneration of Tropical Dry Forest in Madagascar: The Social Institutional Context

**DOI:** 10.1371/journal.pone.0000402

**Published:** 2007-05-02

**Authors:** Thomas Elmqvist, Markku Pyykönen, Maria Tengö, Fanambinantsoa Rakotondrasoa, Elisabeth Rabakonandrianina, Chantal Radimilahy

**Affiliations:** 1 Department of Systems Ecology, Stockholm University, Stockholm, Sweden; 2 Stockholm Resilience Centre, Stockholm, Sweden; 3 Department of Biology and Plant Ecology, University of Antananarivo, Antananarivo, Madagascar; 4 Museum of Art and Archaeology, University of Antananarivo, Antananarivo, Madagascar; University of Pretoria, South Africa

## Abstract

Loss of tropical forests and changes in land-use/land-cover are of growing concern worldwide. Although knowledge exists about the institutional context in which tropical forest loss is embedded, little is known about the role of social institutions in influencing regeneration of tropical forests. In the present study we used Landsat images from southern Madagascar from three different years (1984, 1993 and 2000) and covering 5500 km^2^, and made a time-series analysis of three distinct large-scale patterns: 1) loss of forest cover, 2) increased forest cover, and 3) stable forest cover. Institutional characteristics underlying these three patterns were analyzed, testing the hypothesis that forest cover change is a function of strength and enforcement of local social institutions. The results showed a minor decrease of 7% total forest cover in the study area during the whole period 1984–2000, but an overall net increase of 4% during the period 1993–2000. The highest loss of forest cover occurred in a low human population density area with long distances to markets, while a stable forest cover occurred in the area with highest population density and good market access. Analyses of institutions revealed that loss of forest cover occurred mainly in areas characterized by insecure property rights, while areas with well-defined property rights showed either regenerating or stable forest cover. The results thus corroborate our hypothesis. The large-scale spontaneous regeneration dominated by native endemic species appears to be a result of a combination of changes in precipitation, migration and decreased human population and livestock grazing pressure, but under conditions of maintained and well-defined property rights. Our study emphasizes the large capacity of a semi-arid system to spontaneously regenerate, triggered by decreased pressures, but where existing social institutions mitigate other drivers of deforestation and alternative land-use.

## Introduction

Loss of tropical forests and changes in land-use/land-cover affect climate and environmental change at global scales and are of growing concern worldwide [e.g. 1], [2]. However, estimates of tropical forest loss and changes in land-cover are still uncertain and a 50% margin of error appears possible [1], [3]. Not only are rates of deforestation uncertain, but there is also little knowledge about tropical forest regeneration rates [3]. Achard et al. [1] estimated that at a global level, the annual regrowth area of humid tropical forest is 1 million ha compared with the annual forest loss of 5.8 million ha. In other words, annual regeneration may correspond to roughly 20% of the total area of deforestation in the humid tropics. In spite of this, surprisingly little is known about regeneration in terms of functional aspects of biodiversity and generation of ecosystem services for local and regional human consumption and use [e.g. 4], [5]. The local social institutional context is increasingly emphasized in analyses of drivers of forest cover change [6]–[9], with stable forest cover (i.e. effective protection) often found to be a function of strength and enforcement of local social institutions [9]. Although we have some knowledge about the institutional context in which tropical forest loss is embedded [2], [6], [10], little is known about the role of social institutions (sensu Ostrom [11]) in influencing rates of tropical forest regeneration, especially at the local scale [12].

In Madagascar, the rate of total deforestation has been estimated to be high and thought to be a result of a rapidly growing human population and the use of fire as a farming practice [13]. Recent research has, however, challenged both the dramatic deforestation scenarios [e.g. 14], [15] as well as the conventional view that blames farmers for mismanaging natural resources [e.g. 6], [16]. Current estimates of the remaining wet and moist forest cover are significantly higher than estimates published in the 1980's and recent analyses suggest that the island was only partly covered with forest in pre-human times [13]. Further, recent studies reveal a temporally as well as spatially much more complex relationship between human population densities and forest loss than was previously assumed [13], [16].

The dry forest of the semi-arid south and southwest of Madagascar harbors the highest level of plant endemism in Madagascar with 48% of the genera and 95% of the species endemic [17], [18]. The area is also listed as one of the 200 most important ecological regions in the world [19]. Arid conditions have resulted in a historically less intensive slash and burn agriculture and natural fires are infrequent in this system [17]. Since the early 1970's, the dry forest cover has been reported as declining, principally due to clearing for agriculture, cattle herding, timber harvest and charcoal production [20], [21]. Despite global recognition of the value of the southern dry forest, there have been surprisingly few studies on forest cover changes or effects of anthropogenic impacts [22]. Furthermore, and in contrast to other types of forests in Madagascar there are only a few, small areas of dry forest formally under protection [23]. Informal institutions are, however, playing an important role in southern Madagascar to protect these forest ecosystems [24].

In the present study we used Landsat images from southern Madagascar from three different years (1984, 1993 and 2000) and made a time-series analysis of three distinct large-scale patterns: 1) loss of forest cover, 2) increased forest cover, and 3) stable forest cover. Institutional characteristics underlying these three patterns were analyzed testing the hypothesis that forest cover change is a function of strength and enforcement of local social institutions. Our results corroborate this hypothesis and provide in our view an important contribution to the global discourse on strategies for sustainable tropical forest management involving analyses of the role of different land tenure systems, monitoring and enforcement systems [9].

## Methods

### Study area

**Figure 1:**
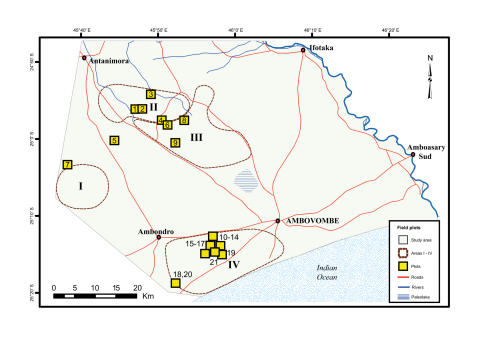
The Androy region is situated in the southernmost part of Madagascar between Lat 24°13′ and 25°24′S and Long 45°20′ and 46°26′ E. Four areas (I–IV) were selected for detailed field investigations. Yellow squares with numbers indicate plots used in ground truthing.

The Androy region is situated in the southernmost part of Madagascar between Lat 24°13′ and 25°24′S and Long 45°20′ and 46°26′E (Fig. 1). The area is characterized by semi-arid climatic conditions with irregular rainfall averaging less than 500 mm per year. The annual rainfall declines from north to south and from northeast to southwest [25]. The dry season usually lasts eight to nine months, between March-October/November, but locally it can extend over several years [14], [26]. The mean temperature is generally between 23 and 26°C but the daily amplitude may be as large as 22.5°C during the cold season, May-October [25].

The dry forest of southern Madagascar is characterized by drought tolerant woody species of Didiereaceae and Euphorbiaceae [18]. The forest is usually 3 to 6 m in height, but emerging trees of the Didiereaceae may reach more than 10 m in height, such as *Alluaudia ascendens* and *A. procera,* the latter dominating forest stands in Central and Northern Androy. In Southern Androy, forest stands are dominated by Euphorbiaceae, mainly *Euphorbia decorsei*. The southernmost part of Androy is a sandy area with paleodunes, which indicate a much more arid climate in the past, possibly at the time of the last glacial maximum around 18.000 BP [27]. The northern part is a hilly upland on Precambrian crystalline bedrock [25].

Settlements in southern sandy areas have longer history and higher population densities ranging from 100–350 people/km^2^ as compared to Northern and Central Androy. The latter areas have historically been used mainly for seasonal grazing, and population densities range from less than 10 to 20 people/km^2^ [28]. The region is dominated by the Tandroy people, primarily known as cattle herders, however cultivation of maize, beans, and sweet potatoes are essential for livelihoods in particular in the south. The direct drivers of forest loss are clearing of land for farming and harvest of fuel wood and timber, either for local consumption or for sale to the urban centers of Ambovombe and Antanimora. In Androy, as in other parts of Madagascar, the traditional land claims inherited from the ancestors (*tanin-drazana*) and which relate to clans and lineages are still effective. This represents a common property regime with collective owners that organize to exclude or regulate non-owners and the extraction of resources [29]. Local institutions such as taboos, have been found to efficiently protect forest patches in Southern Androy [24]. There are few formally protected forest areas in Androy [23]: one National Forest Reserve (Cap Sainte Marie Special Reserve) and two private reserves (Berenty and Kaleta). All three are located outside our study area.

### Analyses of Landsat images

Satellite images from three different years were used for time-series analysis of forest cover changes [see e.g. 30]. The images from 25 June 1984, 15 April 1993 and 28 May 2000 respectively, were all dry season synoptic views from path/row 159/77 with 30 metres resolution. The 1984 and 1993 scenes were Landsat 5 TM and the 2000 scene was a Landsat 7 ETM+. The spectral band combination used for the visual interpretation was 4,5,2 (R,G,B), which means that two near-infrared bands were used in combination with the visible green band. The software ArcInfo (ESRI) was used for the GIS mapping and ERDAS 9.0 (Leica) was utilised for all processing of the satellite images.

Since a classification of a whole Landsat scene covering 180*180 km, would produce considerable error and thereby cause misinterpretations, a clip was made from each scene to the area of interest, approximately 87×63 km = 5481 km^2^ (Lat 24°47′ and 25°24′S, Long 45°36′ and 46°26′E) (Figure 1). Rectification to one pixel accuracy was performed for each sub-scene. An unsupervised classification containing 250 classes was performed for each of the images including all six bands with 30 metre resolution [31]. A preliminary reclassification was based on initial fieldwork in 2001, while the final classification was based on ground truthing carried out in May 2002 and January 2004. From the classified images, only the class for dense/mature forest was extracted for further analysis. A change detection function was applied on the three classified forest images to show changes that had occurred between 1984, 1993 and 2000. The resulting image was thereafter cleaned up with a nibble function and a boundary clean to remove noise. Remaining misclassified areas such as riparian forest and sisal (*Agave* sp.) plantations were removed manually. Within the studied area of 243 600 ha, we calculated based on the classified Landsat images, the total areas for five classes:

1. Forest 84/–/– Forest in 1984 only (loss of forest after 1984)
2. Forest 84/93/– Forest in 1984 and 1993 (loss of forest after 1993)
3. Forest 84/93/00 Forest during 1984, 1993 and 2000 (stable forest)
4. Forest–/93/00 Forest in 1993 and 2000 (increase of forest cover after 1984)
5. Forest–/–/00 Forest in 2000 only (increase of forest cover after 1993)

For comparison and control of the supervised classification an NDVI (Normalised Difference Vegetative Index) analysis was performed using areas representing the three patterns: loss of forest cover, increasing forest cover, stable forest cover. NDVI indices were calculated using the formula NDVI = (NIR-Red)/(NIR+Red) where NIR is the near infrared band 4 and red is the visible red band 3 from the Landsat ETM+image as defined by [32]. This calculation produces a new single layer with pixel values ranging from−1 to 1. The highest values indicates dense vegetation with high amounts of chlorophyll since almost all visible red light is absorbed for photosynthesis whereas the majority of the infrared light is reflected back. When vegetation cover decreases the red wavelength reflectance will increase and infrared reflectance will decrease and the pixel values are approaching zero. Values close to zero indicate bare soil, rock and urbanised areas while values close to−1 indicate clouds or water bodies [31].

### Ground truthing of forest classification

Surveys to verify forest classification were carried out in May 2002 and January 2004. Four areas, corresponding to the three forest classes, were selected for detailed field investigations (Figure 1). The areas were located and mapped using a mobile GIS system, ArcPad from ESRI, running on handheld computers/GPSs. We used survey methods appropriate in each area for validating the classification: a long transect in the area of large loss of forest cover, plot analyses for distinguishing between stable and regenerating forests and transects around sacred forests with access restrictions.

#### Pattern I: Loss of forest cover

In Western Androy (area I in Figure 1) we used an 11 km long transect along a dirt road and estimated: a) dominant species, b) mean height of woody species, and c) the frequency of fields and logging along the transect.

#### Pattern II and III: Increase in forest cover and stable forest cover

In Northern and Central Androy (areas II and III in Figure 1) we used 20×20 m plots to verify the classes of dense stable forest and increasing forest cover. Five plots were located in the area classified as regenerating (area II in Figure 1), and four plots in the area classified as stable and dense forest cover (area III in Figure 1). Among the dense/stable forest plots, two were located in informally protected sacred forests and two plots in unprotected forests (See Table 1). Plots were located randomly within stands of *Alluaudia procera*. In all plots, individual trees and shrubs were identified to species. Each individual's height was estimated using a metered stick. Diameter at breast height (dbh = 130 cm) was measured for all individuals over 150 cm height. In two of the plots, drilled cores were taken from *A. procera* for estimations of number of growing seasons (n = 30–100 individuals per plot) (see Table 1). Tests of statistically significant differences on differences in densities, height and dbh were performed using non-parametric tests (Mann-Whitney U-test). Analyses of species richness in relation to sampling effort using the Chao index [33] revealed that sampling efforts were adequate for the regenerating forest, but in the stable forest variation between plots were large and a larger sample effort needed.

**Table 1:** Ground truthing based on vegetation and demographic analyses in areas I (transect), II, III (plots 20×20 m) and IV (plots and transects see 24).

Area I–Western Androy Loss of forest cover	Area characterized by a mosaic of fields, pastures and shrub lands with low density of woody plants<150 ha^−1^>2 m height. *Alluaudia procera* rare, *A. dumosa* the most common woody species.						
Area II-Northern Androy. Increased forest cover	No of woody species	Height *A. procera* (m) Mean±S.E.	Dbh *A. procera* (cm) Mean±S.E.	Density of *A. procera* ha^−1^ (<2 m)	Total density woody plants ha^−1^ (>2 m)	No of growing seasons* *A. procera*	Density of *C. greveii* ha^−1^ (<2 m height )
**Plot no.**							
1	17	3,6±0,2	6,4±0,4	1275	2125	NA	46
		n = 136	n = 136				
		n = 136	n = 136				
2	20	1,9±0,1	3,1±0,3	2875	875	NA	167
		n = 150	n = 150				
3	19	2,7±0,1	3,9±0,2	3200	2175	NA	549
		n = 215	n = 215				
4	24	3,8±0,3	6,2±0,6	675	1175	NA	2161
		n = 74	n = 74				
5	19	2,4 ±0.08	4,9±0.5	1200	2025	25±1.6 (n = 116)	863
		n = 126	n = 126				
**Mean±S.E.**		**2,9±0,4**	**4,9±0.6**	**1845±500**	**1675±271**		**757±379**
**Area III-Central Androy**							
**Plot no.**							
Stable forest cover 6	14	10,9±0,6	18,0±1,0	25	900	135±7.9 (n = 37)	0
		n = 37	n = 37				
Sacred forests 7	19	6,7±0,3	10,9±0,6	250	2500	NA	389
		n = 110	n = 110				
Other dense forests 8	17	4,5±0,3	5,6±0,4	550	1150	NA	21
		n = 68	n = 68				
9	8	9,0±0,2	9,1±0,2	25	2225	67.5±1.8 (n = 90)	18
		n = 43	n = 43				
**Mean±S.E.**		**7,8±1.4**	**10,9±2.6**	**213±124**	**1694±393**		**107±94**
**Area IV Southern Androy-Stable forest**	**Woody species richness**	**Total density woody plants ha^−1^ (>2 m)**	**Forest patch size (ha)**	**Sampling method (area, m^2^)**			
*Sacred forests*							
10	22	3275	78	transect (128)			
11	28	3550	94	transect (192)			
12	22	2125	2	plot (400)			
13	25	2083	0.6	transect (360)			
14	25	4850	2.3	transect (250)			
15	9	825	1.3	plot (400)			
16	20	1875	<1	plot (400)			
17	16	1225	<1	plot (400)			
**Mean±S.E.**		**2476±470**					
*Public forest*							
18	23	2275	<1	plot (400)			
19	19	2700	<1	plot (400)			
20	20	1400	<1	plot (400)			
21	26	3150	<1	plot (400)			
**Mean±S.E.**		**2381±373**					

* *A. procera* produces clearly visible tree rings, which due to occasional supra-annual periods of draught, may not necessarily correspond to annual growth rings . The cores (0,5 cm in diameter) were drilled at breast height through the center of stems>10 cm dbh using a standard dendrochronology drill.

In the southernmost part of the study area (area IV, Figure 1.) forest distribution is fragmented with forest patches (1–90 ha) scattered in the agricultural landscape. In most of the forest patches (all>5 ha), access is highly restricted due to taboos [24]. Line transects (and where possible 20×20 m plots) were used for floristic analysis of twelve of these forest patches, see [24] and Table 1.

We used a multivariate analysis for validation of the forest classification, i.e. Polythetic Agglomerative Hierarchical Clustering (PAHC) [34] with the software PRIMER v.5. In PAHC a resemblance matrix is computed on standardized data and a hierarchy of increasingly large clusters is analyzed. In our analysis the distance matrix was based on group average linkages. The variables included in the PAHC analysis were: height and diameter of *A. procera*, proportion of juvenile *A. procera* (<2 m height) and abundance estimates of woody species >2 m height. These data were collected from plots 20×20 m in regenerating (n = 5) and dense mature forests (n = 4) in northern central Androy. PAHC is especially well suited for analysis of community similarity/dissimilarity when the purpose of the analysis is mainly descriptive and the sample size is low to moderate (i.e. sampling entities<50, our sample = 36).

### Larger temporal and spatial analyses of forest cover change

To provide a background to recent changes in land cover, we digitized topographical maps from 1955 and 1957 (Foiben-Taosarintanin'i Madagasikara, hereafter referred to as FTM) based on aerial images from the 1950's and extracted areas classified as forest. This was compared with the Landsat images using a change detection analysis. Several sources of error arise when data in different formats are transformed and used in change detection and time-series analyses [cf. 30], [35]. Thus, the comparison has a high degree of uncertainty and the results should be interpreted with caution [see also 13].

To get an estimate of the forest change in the western parts of Madagascar, Landsat TM images from the mid and late 1980s and Landsat ETM+images from 2000 were used. Three images from each period were classified independently using the same approach as for the main study area. This general approach was based on the experience from the classification of the core study area. A bi-temporal analysis was performed to find changes in forest cover which represent rough estimates since no ground truthing was performed.

### Analyses of local social institutions

In the four areas (Figure 1), information on local views on forest cover change, drivers, and local institutions, e.g. the rules-in-use [11] including property right schemes and enforcement characteristics [11], [29], was obtained through interviews. To avoid applying preconceived ideas of local institutions, we used a qualitative interview approach described by e.g. Kvale [36] rather than a predefined questionnaire, and held semi-structures open-ended interviews with a checklist. The checklists included the following questions: a) who has access to forest resources, b) which rules regulate access, c) which authority is responsible for rule enforcement, and d) to what extent are the rules actually followed and enforced. Further, we also discussed with all informants their view on forest cover changes and, drivers of change and how people have responded to temporary drought conditions.

Informants were the two forest officials active in the study area, located in Ambovombe and Antanimora, and key informants as well as other villagers in the four areas. Key informants were persons with authority in relation to forest resources at village level, either as representatives of the official local government, *fanjakana*, village presidents and counselors, or of customary authority, the *fokonolona*, village elders and clan leaders. These were all men of various ages. Official representatives were generally younger than persons representing the customary authority. To supplement and triangulate their opinion, interviews were also held with women and younger persons in the villages. In total, 26 informants were interviewed in ten local communities (Area I: Mareñy, Lahabe, Bemonzola n = 8; Area II: Manave and Mitsoriake n = 8; Area III: Ankilivalo and Belaza n = 3; Area IV: Ambonaivo, Ambazoa, Marolamainty n = 7). The interviews with the forest officials had a broader geographical focus compared to the community interviews, but site-specific questions were asked also to the foresters for triangulation of the interview information from the local key informants.

### Analysis of environmental and socio-economic drivers of forest change

The pattern of forest dynamics was also tested against available data on alternative drivers of forest change that had a spatial resolution relevant for the scale of our analysis. Maps provided by FTM give information on distance to main roads as an indicator of access to markets of forest products. Population censuses in Madagascar do not have the appropriate resolution for our study areas, but the LandScan 2001 Global Population Database (Oakridge, TN: Oak Ridge National Laboratory http://www.ornl.gov/gist/) (spatial resolution 1 km^2^) was nonetheless used to get an estimate of population densities in the four areas.

Local trends in rainfall patterns as a driver of forest change were analyzed using 1) dataset from Systèm d'Alerte Précoce (SAP, Union Européene, Ambovombe, Jan. 2004), 2) published information and 3) oral information through interviews. Long-term reliable local precipitation data do not exist and we were only able to obtain data from three locations within our larger study area, limited to the period 1998–2003 (Area I: Jafaro, Area IV: Ambazoa and Imongy 25°19′S, 45°28′E). The spatial resolution of the data available on precipitation and droughts did not allow for an analysis of the differences between the four areas.

## Results

### Changes in forest cover 1984–2000: ground truthing and analyses of Landsat images

To verify our interpretation of the Landsat images, the ground truthing was based on analyses of forest plots and field surveys in the four selected areas (Fig. 1). Based on these field surveys we defined dense forest as areas with >800 trees ha^−1^ (>2 m height) (Table 1) and we developed field criteria for verifying classification of the three different patterns of forest cover change.

#### Pattern I–Loss of forest cover

**Figure 2:**
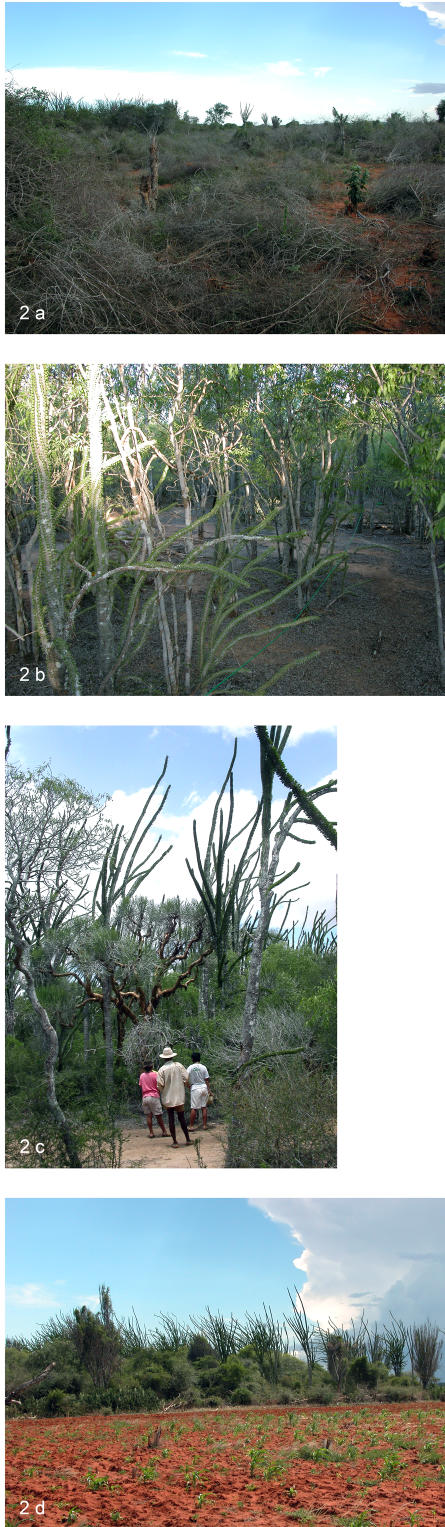
Pictures from the four surveyed areas a) degraded/thinned forest (Area I), b) regenerating forest (Area II), c) stable forest (Area III), d) forest patch protected by local taboos (Area IV). Pictures taken in January 2005.

The surveyed area I was approximately 10 000 ha (Fig. 1) and was visibly directly impacted by human activities on a large scale (Fig. 2a). Along the 11 km transect, we documented numerous (>20) patches that had been cleared for pastures and fields. The surrounding forest was logged and thinned with the mean vegetation height<2 m. *Alluaudia procera,* a characteristic species was absent, the mean density of trees (>2 m) was<150 ha^−1^. The remaining shrub vegetation was dominated by the smaller and less economically valuable *A. dumosa* (Table 1).

#### Pattern II–Increased forest cover

The area classified as regenerating (area II in Fig. 1) and selected for field analysis was approximately 15 000 ha. Plot analysis revealed a high density of juvenile (<2 m height) *A. procera* (mean±s.e. = 1845±500 ha^−1^ , n = 5 plots) and *Cedrelopsis grevei,* (Ptaeroxylaceae) (mean±s.e. = 757±379 ha^−1^, n = 5 plots) (Fig. 2b). Mean number of growing seasons for sampled *A. procera* in one population was estimated to be 25±1.2 (n = 116). Species number of woody plants>1 m height in the regeneration forests was less variable (range 17–24, n = 5 plots) compared to the forest with stable cover (range 8–19, n = 4 plots) (Table 1).

#### Pattern III–Stable forest cover

The areas III and IV were classified as areas with dense, stable forest. In area III, approximately 22 000 ha (Fig. 1 and 2c) the mean density of woody plants (>2 m height) (mean±s.e. = 1694±393 ha^−1^, n = 4) was not significantly different from area II, (Mann-Whitney–test, P>0.05). However, for juvenile (<2 m height) *A. procera* (mean±s.e. = 213±124 ha^−1^ , n = 4) and *C. grevei* (mean±s.e. = 107±94 ha^−1^ , n = 4) density was significantly lower than in area II (Mann-Whitney U-test, P<0.02, P<0.05, respectively, Table 1). Mean height and diameter (dbh) of *A. procera* was significantly larger than in area II (Mann-Whitney U-test, P<0.05 and P<0.02 respectively). Mean number of growing seasons for sampled *A. procera* was estimated between 67.5±1.8 (n = 90) and 135±7.9 (n = 37) in the two populations sampled (Table 1).

**Figure 3:**
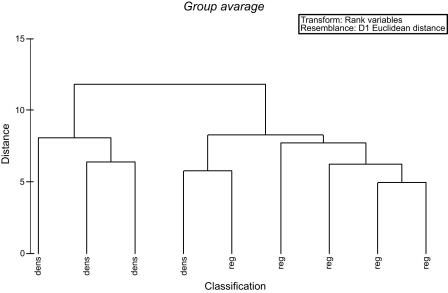
The result of cluster-analyses (PAHC) of vegetation data and demographic data of *Alluaudia procera* from plots 20×20 m in regenerating (n = 5) and dense mature forests (n = 4) in northern central Androy. The distance is based on average group linkages. Variables included in the model: height, diameter, proportion of juveniles (<2 m) of *Alluaudia procera* and abundance estimates of woody species>1 m height. The analysis was used for validation of the forest classification in the Landsat images.

The multivariate analysis (PAHC) validated our forest classification with dense, stable forests (pattern III) and regenerating forests (pattern II) representing two relatively clear distinctive groups (Fig. 3).

In area IV classified as stable forest and located in the southern part of our study area (Fig. 1, approximately 13 000 ha), forest patches ranged in size from<1 ha to over 90 ha (Fig. 2d). A total of 69 patches were larger than 5 ha [24]. Together the forest patches represented approximately 3% of land cover. Twelve of these forest patches were floristically analyzed [24], and forest patch size, species richness and plant density are listed in Table 1. Due to access restriction, sampling was difficult and the floristic information difficult to use in comparison with the other surveyed areas. The density of woody plants were found to be high in both sacred forests (mean = 2476 ha^−1^, n = 8) and public forests (mean = 2381 ha^−1^, n = 4) (Table 1).

**Figure 4:**
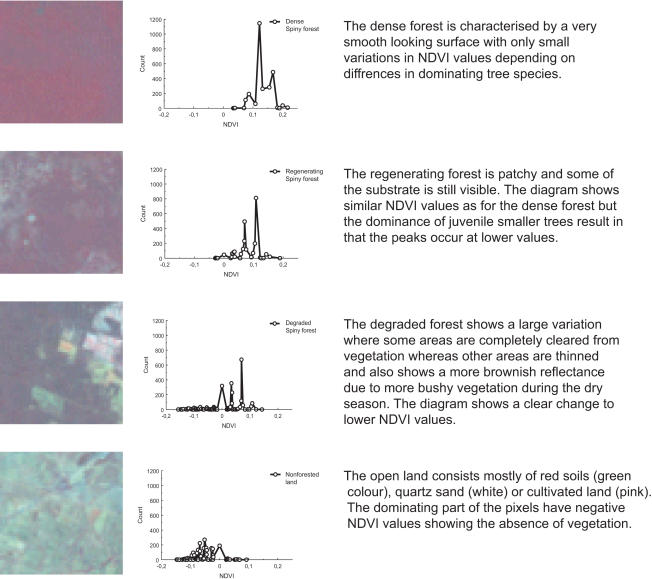
In the NDVI analysis (Normalised Difference Vegetative Index), the dense mature forests show multiple peaks due to differences in species composition, the regenerating forest is separated with lower NDVI values, while degraded forest and open land has distinctly different patterns. The examples given here are subsets (1.5×1.5 km) from the 2000 Landsat 7ETM+image.

The NDVI analysis separated the different forest cover change patterns. The different areas show a range in NDVI values but they all have distinct peaks in the 0 (0 = bare soil) to 0,22 interval indicating differences in dominating plant species (Fig. 4). The overlap is 67% comparing the patterns “Loss of forest cover” and “Stable forest cover”. The overlap between patterns “Increased forest cover” and “Stable forest cover” is 91%. NDVI values increase with increased forest cover, but the range of values decreases. The dense forests show multiple peaks due to differences in species composition, the regenerating forest is separated with lower NDVI values (Fig. 4).

**Figure 5:**
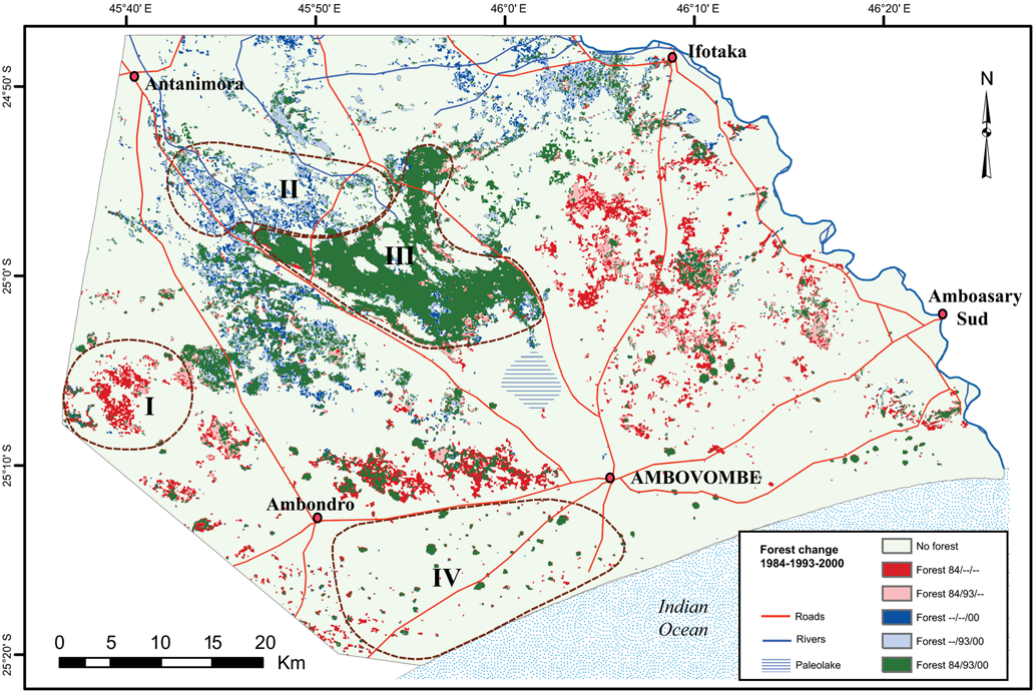
Time-series analysis of changes in forest cover based on satellite images (dry season synoptic views from 25 June 1984 (Landsat 5 TM), 15 April 1993 (Landsat TM) and 28 May 2000 (Landsat 7 ETM+) from Androy, southern Madagascar. Three patterns of forest cover change between 1984 and 2000 is presented: 1) red-reddish areas-loss of forest, 2) blue-bluish areas–increase of forest cover and 3) green areas–stable forest.

With the final classification validated by ground truthing and the NDVI analysis, we calculated total changes in forest cover in the study area. In the 1984 Landsat image, 41 500 ha of the studied area was classified as being covered with dense, mature forest. In 1993, 30 284 ha was classified as having a similar dense forest cover and 11 259 ha had been degraded/thinned (Fig. 5). On the other hand, an area corresponding to 6 813 ha not classified as dense forest in 1984, had regenerated. In 2000, 25 449 ha was classified as dense forest. In comparison with 1993, there was a decrease of 4 835 ha of dense forest, but at the same time regeneration by 6 255 ha from 1993 (Fig. 5).

**Figure 6:**
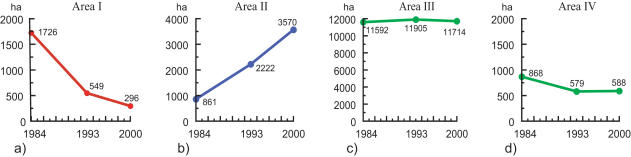
Changes in forest cover (ha) in the four surveyed areas (Area I–IV) based on analyses of Landsat images from 1984, 1993 and 2000.

In our four surveyed areas, area I showed a marked decrease of forest cover from 1726 ha in 1986 to only 296 ha in 2000 (−83%) (Fig. 6). Area II showed instead a rapid regeneration from 861 ha in 1986 to 3570 ha in 2000 (+417%). Areas III and IV showed changes between the same years from 11 592 ha to 11 714 ha (+1%) and 868 ha to 588 ha respectively (−32%) (Fig. 6).

For the total area studied, the net result was that dense forest and regenerated forest combined made up 37 103 ha in 1993 giving an 11% decrease (4397 ha) from 1984 with a mean rate of decrease of 489 ha/year. The net result for 2000 was that dense forest and regeneration in total amounted to 38 523 ha, giving an increase of forest cover of 4% (1420 ha) from 1993 with an average rate of increase of 203 ha/year. The overall result between 1984 and 2000 was a total decrease of 7% of total forest cover (Fig. 5).

In the comparison with topographical maps, representing the interval from 1950 to 1984, we estimated the forest cover loss to ca. 65%, the cover being reduced from 154 100 ha in 1950 to 54 200 ha in 1984 (average rate = 2700 ha/year). In the analysis of the larger area of southern Madagascar west of Androy, the same northern limit was used as for the core study area in Androy. In the late 1980s the western area, comprising 9100 km^2^, was covered by 1768 km^2^ (19%) dry forest, mainly *Alluaudia comosa*. By 2000 the forest cover had decreased to 717 km^2^ (8%) giving an overall decrease of the forest of 59% (4800 ha/year).

### Analyses of local social institutions

Interviews revealed clear differences in the types and functioning as well as efficiency of local institutions regulating use of and access to forest resources in the four areas, as summarized in Table 2. In all four areas, formal control as represented by the district office of water and forest had very limited power over use of forest resources, a view communicated by local leaders as well as the forest officials themselves.

**Table 2:** Institutional characteristics of the four surveyed areas.

Area	Type of forest cover change	Area surveyed (km^2^)	Ind./km^2^ 1	Institutional characteristics^2^	Social-ecological interactions–effects on forest cover
I Western Androy	Loss of forest cover (pattern I)	100	20	Neither formal nor customary tenure enforced. Rules for forest access and utilization	Open access conditions leading to land clearings to acquire land coinciding with increasing aridity and recurrent droughts.
II Northern Androy	Increased forest cover (pattern II)	150	<10	Well defined customary land rights. Limited capacity for monitoring and enforcement due to low population density.	Decline in grazing pressure due to permanent and temporal migration of people and decline in seasonal grazing in the area. Decline in land clearings for agriculture.
III Central Androy	Stable forest cover (pattern III)	220	<10	Well defined customary land rights. Limited capacity for monitoring and enforcement due to low population density.	Decline in grazing pressure due to permanent and temporal migration of people and decline in seasonal grazing in the area.
IV Southern Androy	Stable forest cover (pattern III)	130	>150	Well defined customary land rights, strengthened by taboos preventing forest resource extraction, that are very well monitored and enforced.	Changes in seasonal migration with cattle (longer duration, more distant).

1 Source: (50)

2 Interviews with forests officers and local key informants. See also (22).

#### Pattern I: Loss of forest cover

In the surveyed area (Area I), neither formal nor customary tenure are properly enforced. Western Androy has a history of abandonment and recolonization during the last century. The group with the ancestral land rights migrated elsewhere during early 19^th^ century. The present inhabitants settled around the 1950's, and in an analysis of aerial photos from this period, the forest was described as regenerating in abandoned fields [37]. During the droughts of the 1980's, illegal land claims and clearings of land have been common in the area (Forest officer Antanimora pers. comm.). The customary property right system authorized by clan elders is not in place and forest has been cleared to seize land in an unregulated competition with others (Forest Officer in Antanimora, pers. comm.). Several recent clearings and signs of extensive cutting were observed in May 2003 and January 2004. Informants in different parts of Androy confirmed that clearing land is seen as a way to gain formal rights to land [cf. 15]. According to local leaders, a customary practice is used to claim land, using cacti (*Opuntia* spp) to mark new borders, but the institutional mechanisms that regulate the practice are not in use, as the elders among the new inhabitants have no established authority over the land. In practice, there is an open access situation, with insufficient enforcement of formal rules and a malfunctioning customary system.

#### Pattern II and III: Increased and stable forest cover

In Northern (Area II) and Central (Area III) Androy, settlements are small and scattered and the forest is utilized as a seasonal resource for cattle herding by people from the more densely populated south and southwest. In contrast to Western Androy, customary land rights are still actively in place. For example, seasonal dwellers must have permission from the clan authorities. Also, recent permanent settlers are dependent of the original inhabitants that have the ancestral rights. In two villages, informants reported that permission to cut trees is required from formal (*fanjakana*) as well as customary authorities *(fokonolona)*. However, population densities are low (Table 2) and informants reported difficulties in controlling the behavior of outsiders in the forests. One village had approached the forest officer in Antanimora for assistance to control illegal harvesting of forest resources.

#### Pattern III: Stable forest cover

In Southern Androy (Area IV) the tenure of each parcel of forest is very well defined, and all forest patches larger than 5 ha, as well as many of the smaller ones, have been identified as taboo forests belonging to a clan or a lineage [24]. Traditional beliefs and taboos appear to effectively restrict the entrance to, and the use of any resources from the forest patches [24].

### Analysis of socio-economic and environmental drivers of forest change

**Figure 7:**
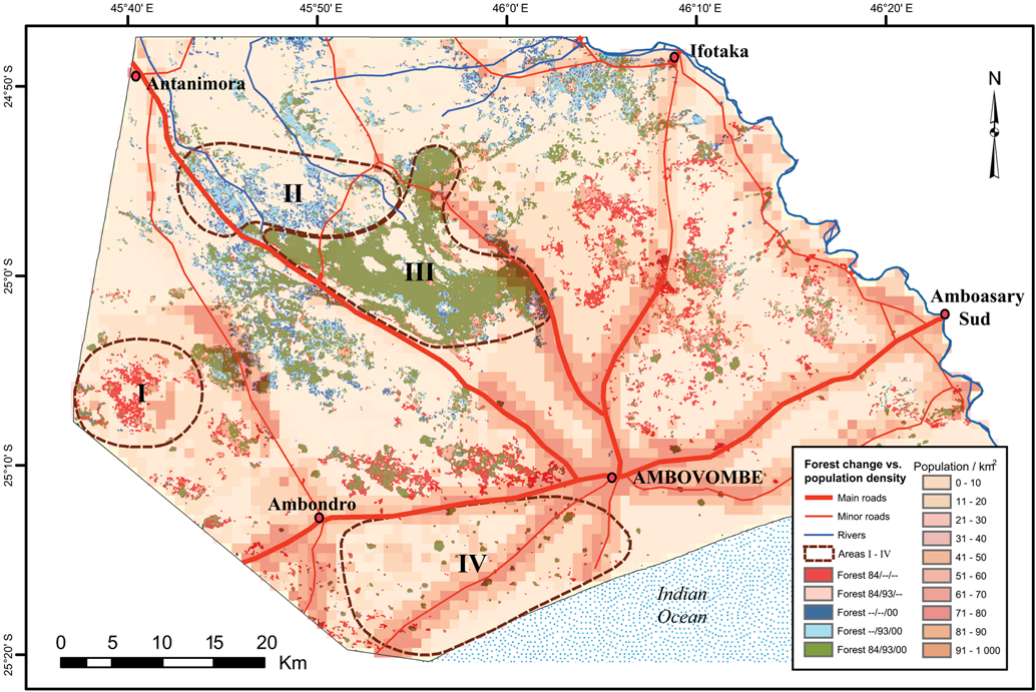
The four surveyed areas and estimates of human population density and distances to main roads as indicator of access to markets of forest products. Population censuses based on LandScan 2001 Global Population Database (Oakridge, TN: Oak Ridge National Laboratory http://www.ornl.gov/gist/). Map source: Foiben-Taosarintanin'i Madagasikara (FTM)

**Figure 8:**
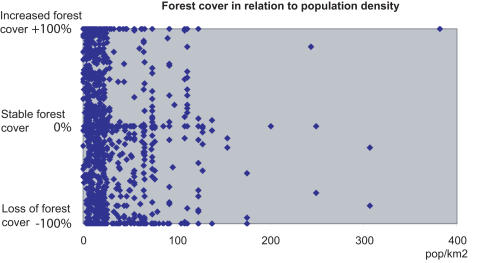
The relationship between forest cover change (%) during 1984–2000 in Androy, and human population density Population censuses based on LandScan 2001 Global Population Database (Oakridge, TN: Oak Ridge National Laboratory http://www.ornl.gov/gist/). The analysis was based on 1 km^2^ plots (n = 564) throughout the area covered with forest either in 1984 or 2000.

Human population density and market accessibility (proximity to roads) did not relate in a simple way with forest cover change (Fig. 7 and 8). Western Androy (Area I) had the highest rate of forest loss, yet poor connections with regional markets for forest products. Distance to major routes is>20 km, whereas comparatively well served routes pass through Northern, Central and Southern Androy (Fig. 7). Furthermore, in Southern Androy (IV) where forest cover was stable, the pressure on remaining forests resources is high with population densities>150 persons/km^2^ (Table 2, Fig. 7)

**Figure 9:**
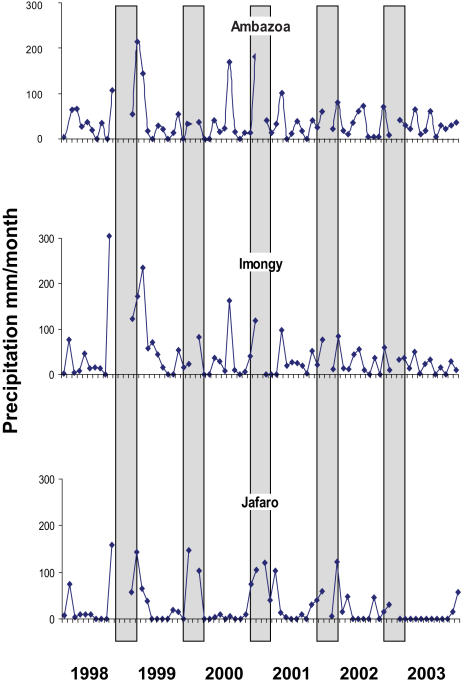
Precipitation (mm/month) during 1998–2003 from three areas in Androy (Area I: Jafaro, Area IV: Ambazoa and Imongy 25°19′S, 45°28′E). (SAP, European Union, Ambovombe, Jan. 2004, see [28]). The gray bars indicate the main cropping season.

Livelihood insecurity associated with recurrent drought periods are a serious concern for the inhabitants in Androy. In our analysis based on the limited availability of precipitation data from our studied sites, there is a trend of decreasing precipitation over time although there are large local variations (Fig. 9). Southern Madagascar has experienced declining precipitation since the 1970's and recurrent drought conditions since 1981, which have almost become chronic [15]. Severe droughts in southern Madagascar have been reported in 1981, 1988, 1990, 1992, 2000 and 2003, the most severe in 1981 affecting one million people, in 1992, 950.000 and in 2003, 600.000 people (EM-DAT: The OFDA/CRED International Disaster Database www.em-dat.net). As a response to the periods of drought, migration to areas and urban centers outside Androy has increased during the last decades [e.g. 15].

In interviews, local climatic factors were found to have important impacts on land use decisions in Androy. Declining and/or more erratic rainfall have resulted in farming becoming a less reliable source of livelihood, and the number of applications for clearing new farmland had declined significantly during the last five years (Forest Officer in Antanimora, pers. comm.). On the other hand, the pressure on forest commodities that could generate an income such as timber or fuel wood, increased (ibid.). Droughts exert strong pressure on livelihoods in all four studied areas, but the vulnerability of the local communities varied as well as their responses (Table 2). In Northern Androy (Area II), informants reported that the severe droughts in 1982 and 1990–1992 caused death among both people and cattle and extensive migrations elsewhere. The droughts have also caused alterations in the patterns of seasonal migrations, as zebu herds are moved much further north to find water and are kept away for longer periods of time than what was previously the custom (G. Heurtebize pers. com.), thus alleviating the pressure on the forests in areas II and III.

## Discussion

We detected a marginal change in total forest cover in Androy over the recent 15 year period 1984–2000, contrasting with other reports of deforestation being as high, or higher than, in the eastern moist forests [e.g. 21], [38], [39]. On the other hand, large-scale reduction of forest cover appears to have occurred in the rest of southwestern Madagascar during the same time period (loss of 59% forest cover) and in Androy during the period 1950–1984 with an estimated 65% reduction. Although the absolute figures for these deforestation rates should be interpreted with caution, our analyses nonetheless suggest that for Androy as a whole, a marked reduction in the average annual rate of forest cover loss occurred from 1984–2000. Furthermore, from 1993–2000 we detected a 4% net increase of forest cover with large areas having high abundance of young vigorously growing trees. This observation contrast the common assertion that dry tropical forests have a low regeneration potential [e.g. 21], [38], [40]. In our study areas, most of the loss of forest cover occurred during the period 1984–1993 compared to the period 1993–2000 (Fig. 6), while regeneration seems to have occurred at similar rates during the two periods (Fig. 6).

### Forest dynamics in a local institutional context

Contrary to the common assumption about drivers of forest change, deforestation was most rapid in an area with low population density and relatively long distances to markets (Area I) as indicated in Fig. 7. A stable forest cover was found in the area with highest population density and good market accessibility (Area IV) and stable and increases of forest cover also occurred in areas with low human population densities (Area II) (cf. discussion in [41]).

Our hypothesis that the strength and enforcement of local institutions are important in determining patterns of forest cover change was largely corroborated by our results. The largest forest reduction in our surveyed area occurred in an area with distinctly insecure property rights and an open access situation. Stable forest occurred in areas where property rights are well defined and strong social institutions and functioning enforcements are present. Furthermore, the transition to regeneration forest occurred in areas where property rights are still well defined and local norms and rules are enforced. These findings correspond well with insights on functioning institutions, whether government-controlled, private property or common property regimes and forest conditions [9]. Our data indicate that even over distances less than 50 km, local institutions may differ in their capacity to mediate environmental drivers of forest cover change. This illustrates the role of institutions and social-ecological interactions at microscale.

Monitoring and enforcement of rules are costly functions in conservation and forest management [7], [8]. Gibson et al. [42] argue, based on an extensive set of case studies, that regular monitoring and sanctioning of rules is a necessary condition for successful resource management. Our findings support this, and highlight the role of present local communities in monitoring and sanctioning human behavior in relation to forest resources. Kaufman and Tsirahamba [43] pointed out that mobile pastoralists in southern Madagascar have a strong tradition of keeping large forest areas as a reserve for livestock during times of stress. Our study emphasizes that in northern Androy, with stable or increasing forest cover, such rules-in use for forests management are still in place. Local informants claimed, however, that in areas of low population density, they were difficult to enforce. To our knowledge, no previous study has presented empirical data on forest regeneration in a local institutional context [cf. 12].

Compared to other African countries, Madagascar has an innovative legislation for governance of forest resources, the GELOSE, Gestion Locale Sécurisée, from 1996, that allows for a transfer of some management rights to local communities [44]. Also, the recent scheme for expanding areas protected for biodiversity conservation in Madagascar, knows as the “Durban Vision”, emphasizes reserves that allow human settlements and resource use [23].

Changes in precipitation expressed as periodic droughts have affected the whole region, and since 1982 insufficient rainfall has led to temporary famines particularly during 1990–92 and during 2003. Such events have resulted in declines in livestock numbers and grazing intensity [cf. 45] but the effect differs between the surveyed areas. The droughts in 1982 and 1990–92 had particularly severe impacts in the regeneration area, causing high mortality among livestock as well as large-scale migration of people [cf. 15]. We estimated, based on growth ring analysis that the majority of trees (>10 cm at dbh) in the regeneration area have established within the last 20–30 years, thus coinciding with the period of reductions in human population density and grazing pressure.

Patterns of forest regeneration, in particular in dry tropical forests, are often neglected in both research and policy development [cf. 7]. The few studies that have identified large scale forest regeneration demonstrate how migration and land abandonment create space for forest renewal [5], [12], [46]. Also in our study, forest regeneration in Northern Androy was associated with migration and declining livestock pressure. On the other hand, as found in Western Androy, previous migration and abandonment contributed to a situation with insecure property rights, non-functioning monitoring and enforcement of local rules, and degrading forest. Also, southwestern Madagascar has experienced large reduction in forest cover during the last 20 years which have been attributed at least partly to migration and agricultural expansion in areas with insecure property rights [14], [43]. Similar processes of open access situations created by migration have been reported in other parts of Madagascar and Africa [15], [39], [47], [48].

The need to go beyond preconceived ideas regarding relationships between people and forest dynamics has been clearly illustrated [6], [49], [50]. The expansion of human settlements is often seen as a serious threat to biodiversity conservation [51]. An emerging literature points to the capacity of local communities to develop flexible governance systems that are responsive to local ecosystem dynamics [10], [52], [53] that is often overlooked. More research on the dynamics of local institutions and under which conditions functioning local governance of ecosystems can develop is needed. Failure to recognize existing institutions at the local scale, as well as their capacity for flexibility and self-organization, risks the overruling and loss of functioning mechanisms for sustainable forest governance [24].

### Dry tropical forest regeneration-implications

Rapid recovery of vegetation in semi-arid areas, as observed in this study, has elsewhere been observed in situations of changing precipitation and grazing pressure falling below a low critical value [54]. Rather than gradual responses to changing conditions, semi-arid systems may experience sudden transitions from one state to another triggered by e.g. management and climatic conditions [54], [55]. Previous theoretical analyses of arid and semi-arid ecosystems and degradation have emphasized that human population growth and over-grazing have lead to a degradation process moving these ecosystems away from a natural single equilibrium state [e.g. 56]. This view has been increasingly challenged and recent studies have emphasized that semi-arid systems exhibit large spatial and temporal variations and are far better described in terms of non-equilibrium systems [e.g. 54], [57] with different alternative states [54].

Large-scale regeneration of tropical forests represents an important economic potential as well as a potential for conservation of biodiversity [e.g. 58], [59] and carbon sink services [60]. In a review, Guariguata and Ostertag [61] concluded that regeneration capacity in general was high if propagule sources were close by and land use before abandonment not severe. Pascarella et al. [62] and Grau et al. [5] found that forest structure and species richness in secondary successions tended to be similar to mature forests after 25–40 years [see also 4], while some legacies of previous land use activities (e.g. increased large-scale floristic homogenization) could persist for centuries [see also 63]. Direct counts of species of woody plants revealed a high capacity by plant species to disperse and colonize, with the two dominant species in the regeneration area, *A. procera* and *C. grevei,* both belonging to endemic plant genera. However, little is known about the extent to which recovery of species richness reflects overall restored ecological functions and ecosystem services [64]. We also have a poor understanding of the historical pattern of regeneration and loss of forest cover. The regeneration pattern we have observed may be just one out of many deforestation/regeneration cycles repeated during the millennium-long period of human presence in the area [cf. 65].

### Conclusions

The failure to acknowledge forest transitions such as regeneration of forest [66] leads to a linear view of change and misinterpretations of the conditions for sustainable forest management. In spite of being an ecosystem of essential importance for people in Madagascar as well as in other parts of the world, we still have limited understanding of dry forest dynamics and its resilience to different patterns of human use. Our study points to the large, but often neglected, capacity of a dry tropical forest to spontaneously regenerate given a window triggered by declining pressures. We argue that spontaneous forest regeneration can not be understood as an ecological process alone; it is also embedded in an institutional context and critically dependent on functioning local social institutions mitigating drivers of deforestation and alternative land use [cf. 43].

## References

[1] Achard F, Eva HD, Stibig HJ, Mayaux P, Gallego J (2002). Determination of deforestation rates of the world's humid tropical forests.. Science.

[2] Lambin E, Geist HJ, Lepers E (2003). Dynamics of land-use and land-cover change in tropical regions.. Annual Review of Environmental Resources.

[3] Watson R, Noble IR, Bolin B, Ravindranath NH, Verardo DJ (2000). Land use, land use changes and forestry..

[4] Dunn RR (2004). Recovery of faunal communities.. Conservation Biology.

[5] Grau HR, Aide TM, Zimmerman JK, Thomlinson JR, Helmer E (2003). The ecological consequences of socioeconomic and land-use changes in postagriculture Puerto Rico.. Bioscience.

[6] Lambin EF, Turner BL, Geist HJ, Agbola SB, Angelsen A (2001). The causes of land-use and land-cover change: moving beyond the myths.. Global Environmental Change-Human and Policy Dimensions.

[7] Barrett CB, Brandon K, Gibson C, Gjertsen H (2001). Conserving tropical biodiversity amid weak institutions.. Bioscience.

[8] Dietz T, Ostrom E, Stern PC (2003). The struggle to govern the commons.. Science.

[9] Ostrom E, Nagendra H (2006). Insights on linking forests, trees, and people from the air, on the ground, and in the laboratory.. Proceedings of the National Academy of Sciences of the United States of America.

[10] Gibson C, McKean MA, Ostrom E (2000). People and Forests.. Communities, institutions and governance.

[11] Ostrom E (1990). Governing the Commons.. The evolution of institutions for collective action.

[12] Tucker CM, Munroe DK, Nagendra H, Southworth J (2005). Comparative spatial analyses of forest conservation and change in Honduras and Guatemala.. Conservation and Society.

[13] McConnell W (2002). Madagascar: Emerald Isle or Paradise lost?. Environment.

[14] Richard AF, Dewar RE, Schwartz M, Ratsirarson J (2002). Life in the slow lane? Demography and life histories of male and female sifaka (*Propithecus verreauxi verreauxi*).. Journal of Zoology.

[15] Casse T, Milhoj A, Ranaivoson S, Randriamanarivo JR (2004). Causes of deforestation in southwestern Madagascar: what do we know?. Forest Policy and Economics.

[16] Kull CA (2004). Isle of fire.. The political ecology of landscape burning in Madagascar.

[17] Koechlin J, Battistini R, Richard-Vindard G (1972). Flora and vegetation of Madagascar.. Biogeography and ecology in Madagascar.

[18] Rabesandratana R, Jolly A, Oberlé P, Albignac R (1984). Flora of the Malagasy southwest.. Madagascar.

[19] Olson DM, Dinerstein E (2002). The Global 200: Priority ecoregions for global conservation.. Annals of the Missouri Botanical Garden.

[20] Sussman RW, Rakotozafy A (1994). Plant Diversity and Structural-Analysis of a Tropical Dry Forest in Southwestern Madagascar.. Biotropica.

[21] Sussman RW, Green GM, Porton I, Andrianasolondraibe OL, Ratsirarson J (2003). A Survey of the Habitat of Lemur catta in Southwestern and Southern Madagascar.. Primate conservation.

[22] Elmqvist T (2004). The forgotten dry forest of southern Madagascar.. Plant Talk.

[23] Fenn M, Goodman SM, Benstead JP (2003). The spiny forest ecoregion.. The natural history of Madagascar.

[24] Tengö M (2004). Management practices for dealing with uncertainty and change-Social-ecological systems in Tanzania and Madagascar [PhD-thesis]..

[25] Battistini R, Richard-Vindard G (1972). Biogeography and ecology in Madagascar..

[26] Dewar RE, Wallis JR (1999). Geographical patterning of interannual rainfall variability in the tropics and near tropics: An L-moments approach.. Journal of Climate.

[27] Clark CD, Garrod SM, Pearson MP (1998). Landscape archaeology and remote sensing in southern Madagascar.. International Journal of Remote Sensing.

[28] SAP (2002). Données structurelles concernant la sécurité alimnetaire..

[29] Hanna S, Folke C, Mäler K-G (1996). Rights to nature..

[30] Cohen WB, Goward SN (2004). Landsat's role in ecological applications of remote sensing.. Bioscience.

[31] Lillesand TM, Keifer RW (1994). Remote sensing and image interpretation..

[32] Rouse JWJ, Haas RH, Deering DW, Schell JA, Harlan JC (1974). Monitoring the Vernal Advancement and Retrogradation (Green Wave Effect) of Natural Vegetation. NASA/GSFC.

[33] Henderson P (2003). Practical methods in Ecology..

[34] McGarigal K, Cushman S, Stafford S (2000). Multivariate statistics for wildlife and ecology research..

[35] Turner IM, Corlett RT (1996). The conservation value of small, isolated fragments of lowland tropical rain forest.. Trends in Ecology&Evolution.

[36] Kvale S (1996). Interviews: an introduction to qualitative research interviewing..

[37] Heurtebize G (1986). Histoire des Afomarolahy (Extrême-Sud de Madagascar)..

[38] Sussman RW, Green GM, Sussman LK (1994). Satellite Imagery, Human-Ecology, Anthropology, and Deforestation in Madagascar.. Human Ecology.

[39] Seddon N, Tobias J, Yount JW, Ramanampamonjy JRM, Butchart S (2000). Conservation issues and priorities in the Mikea Forest of south-west Madagascar.. Oryx.

[40] Green GM, Sussman RW (1990). Deforestation history of eastern rain forests of Madagascar from satellite images.. Science.

[41] Locatelli B, Boissau S, Weber J, D B (2004). Does population growth affect wooded-cover dynamics?. Beyond Tropical Deforestation: UNESCO/CIRAD.

[42] Gibson CC, Williams JT, Ostrom E (2005). Local enforcement and better forests.. World Development.

[43] Kaufmann J, Tsirahamba S (2006). Forests and Thorns: Conditions of Change Affecting Mahafale Pastoralists in Southwestern Madagascar.. Conservation and Society.

[44] Bertrand A (1999). La gestion contractuelle, pluraliste et subsidiaire des ressources renouvelables a Madagascar (1994–1998).. African Studies Quarterly.

[45] Raharison L-JR (1997). Effets et conséquences sur le régime des aquifères des anomalies climatiques dans l'extreme sud de Madagascar: cas du bassin endoréique d'Ambovombe.. Cachiers Sécheresse.

[46] Pascarella JB, Aide TM, Serrano MI, Zimmerman JK (2000). Land-use history and forest regeneration in the Cayey Mountains, Puerto Rico.. Ecosystems.

[47] Sheperd G (1992). Managing Africas' tropical dry forests.. A review of indigenous methods..

[48] Horning NR, Goodman SM, Benstead JP (2003). How rules affect conservation outcomes.. The natural history of Madagascar.

[49] Fairhead J, Leach M (1996). Misreading the African landscape; Society and ecology in a forest-savanna mosaic..

[50] Tiffen M, M M, Gichuki F (1994). More people less erosion: Environmental recovery in Kenya..

[51] Luck GW, Ricketts TH, Daily GC, Imhoff M (2004). Alleviating spatial conflict between people and biodiversity.. Proceedings of the National Academy of Sciences of the United States of America.

[52] Berkes F, Folke C (1998). Linking Social and Ecological Systems. Management Practices and Social Mechanisms for Building Resilience..

[53] Berkes F, Colding J, Folke C (2003). Navigating Social-Ecological Systems: Building Resilience for Complexity and Change..

[54] Holmgren M, Scheffer M (2001). El Niño as a window of opportunity for the restoration of degraded arid ecosystems.. Ecosystems.

[55] Westoby M, Walker B, Noy-Meir I (1989). Opportunistic management for rangelands not at equilibrium.. Journal of Range Management.

[56] Swift J, Leach M, Mearns R (1996). Desertification: Narratives, winners&losers.. The lie of the land: Challenging received wisdom in African environmental change.

[57] Leach M, Mearns R, Scoones I (1999). Environmental entitlements: Dynamics and institutions in community-based natural resource management.. World Development.

[58] Bawa KS, Seidler R (1998). Natural forest management and conservation of biodiversity in tropical forests.. Conservation Biology.

[59] Chazdon RL (1998). Tropical Forests–Log 'Em or Leave 'Em?. Science.

[60] Ramirez OA, Carpio CE, Ortiz R, Finnegan B (2002). Economic value of the carbon sink services of tropical secondary forests and its management implications.. Environmental&Resource Economics.

[61] Guariguata MR, Ostertag R (2001). Neotropical secondary forest succession: changes in structural and functional characteristics.. Forest Ecology and Management.

[62] Pascarella JB, Aide TM, Serrano MI, Zimmerman JK (1999). Land use history and forest regeneration in the Cayey Mountains, Puerto Rico.. Ecosystems.

[63] Brown KA, Gurevitch J (2004). Long-term impacts of logging on forest diversity in Madagascar.. Proc Natl Acad Sci.

[64] Silver WL, Brown S, Lugo AE (1996). Effects of changes in biodiversity on ecosystem function in tropical forests.. Conservation Biology.

[65] Willis KJ, Gillson L, Brncic TM (2004). How “virgin” is virgin rainforest?. Science.

[66] Rudel TK, Coomes OT, Moran E, Achard F, Angelsen A (2005). Forest transitions: towards a global understanding of land use change.. Global Environmental Change-Human and Policy Dimensions.

